# Cryo‐EM Structures Reveal Key Mechanisms of Noradrenaline Transporter

**DOI:** 10.1002/mco2.70188

**Published:** 2025-04-14

**Authors:** Peng Su, Mao Li, Fangfang Zhou

**Affiliations:** ^1^ School of Medicine Hangzhou City University Hangzhou China; ^2^ Key Laboratory for Diagnosis and Treatment of Upper Limb Edema and Stasis of Breast Cancer Zhejiang Provincial People's Hospital, Hangzhou Medical College Hangzhou China; ^3^ Institutes of Biology and Medical Science Soochow University Suzhou China

1

In a recent article in *Nature*, Hu et al. [1] unveiled cryo‐EM (cryo‐electron microscopy) structures of the human noradrenaline transporter (NET) in multiple states—unbound (*apo)*, substrate‐bound (noradrenaline), and bound with several clinically used drugs (Figure 1A). These structures, captured in inward‐ and outward‐facing conformations, shed light on the substrate transport and inhibition mechanisms of NET.

**FIGURE 1:**
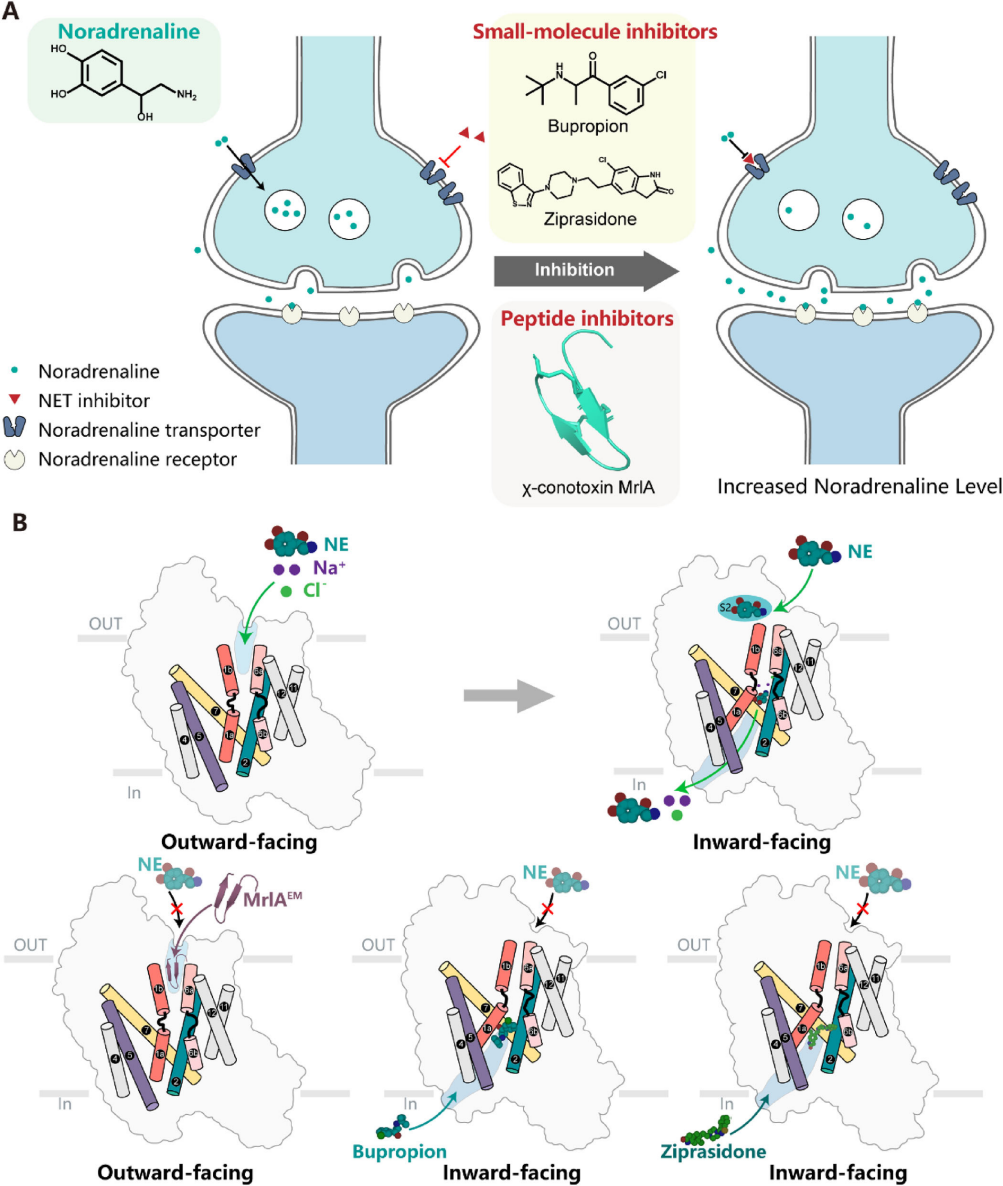
Transport and inhibition mechanisms of noradrenaline transporter (NET). (A) Schematic representation of the working mechanism of the noradrenergic synapse. (B) Schematic representation of the noradrenaline transport cycle highlighting the conformational transitions from outward‐facing to inward‐facing states. The distinct binding modes of various inhibitors are shown, illustrating their interactions with NET during the transport cycle.

The noradrenaline system, dependent on noradrenaline as its primary neurotransmitter, regulates essential physiological functions, including mood, pain perception, sleep‐wake cycle, arousal, attention, feeding behavior, and fight‐or‐flight responses [2]. Noradrenaline system dysfunction is implicated in several mental disorders. NET, located in presynaptic neurons, mediates noradrenaline reuptake, effectively terminating synaptic signaling and modulating neurotransmitter levels. Given the crucial role of NET in maintaining noradrenaline homeostasis, it has emerged as a primary target for the treatment of mental health disorders, including depression, attention deficit hyperactivity disorder, and neuropathic pain. Clinically used drugs exert their effects by targeting NET. However, the detailed molecular mechanisms underlying the effects of these inhibitors remain unclear. In addition, fundamental questions regarding the transport mechanism of NET, such as ligand and ion coupling and the conformational transition across functional states, remain elusive.

To elucidate the mechanisms underlying NET transport and inhibition, Hu et al. [1] expressed human NET and reconstituted it into nanodiscs to comprehensively replicate the native membrane environment. Using cryo‐EM, they obtained structures at 2.6 Å of NET in *apo* and noradrenaline‐bound states, capturing the transporter in inward‐ and outward‐facing conformations. NET operates as a secondary active transporter that leverages electrochemical gradients of sodium and chloride ions. The high‐resolution maps facilitated the precise localization of sodium and chloride ion‐binding sites, highlighting their roles in the transport cycle of NET. In addition to the primary binding site, Hu et al. [1] identified a secondary substrate‐binding site within the extracellular cavity that existed only in the inward‐facing conformation. Upon mutating key residues, including R301 and E382, which interact with noradrenaline at this site, they observed a partial reduction in the transport activity for the E382A mutant, whereas the R301A mutation did not appear to affect transport activity. This finding suggests that the secondary site is complementary in modulating substrate dynamics, potentially influencing the efficiency and regulation of noradrenaline transport under physiological conditions. By resolving the structure of NET in both outward‐ and inward‐facing conformations, they mapped the structural rearrangements essential for its transport cycle. They observed substantial displacement in the core transport domains—transmembrane helices TM1 and TM6—during the transition from the outward to inward‐facing states. This conformational shift also alters the coordination of ions. Notably, the Na_2_ site, which is coordinated by five residues in the outward‐facing state, is reduced to three residues in the inward‐open state, aligning with the sodium release upon cytoplasmic access. This structural insight revealed how ion gradients contribute to substrate translocation, providing a comprehensive understanding of the molecular mechanisms driving NET transport.

In addition to the substrate‐bound structure, Hu et al. investigated the interaction of NET with a potent inhibitor, the conotoxin χ‐MrlA. An analog of this 13‐residue peptide, XEN2174, showed analgesic effects in clinical studies. Notably, χ‐MrlA is the only known non‐competitive inhibitor of NET and selectively inhibits NET without affecting other transporters in the same family, including the dopamine transporter (DAT) and serotonin transporter (SERT). Although resolving the structure of the χ‐MrlA‐NET complex was initially challenging, the authors overcame this problem by using high‐affinity mutants. The resulting structure revealed that χ‐MrlA stabilized NET in the outward conformation through an extensive interaction network. Combined with the mutation study in the [3H] noradrenaline transport assay, this study revealed the critical residues, including F472 in DAT and K470 in SERT, responsible for the selectivity of χ‐MrlA for NET over DAT and SERT, providing a foundation for designing peptide inhibitors of other transporters in the same family.

Selective serotonin reuptake inhibitors (SSRIs) and serotonin–norepinephrine reuptake inhibitors (SNRIs) are commonly prescribed for major depressive disorder. Despite their effectiveness and safety, approximately 40%–60% of patients do not achieve complete symptom remission with these medications. Moreover, the long‐term use of SSRIs and SNRIs is often associated with adverse side effects, such as sexual dysfunction and weight gain, which can considerably impact patient compliance and overall quality of life. These limitations highlight the need for alternative treatments with improved efficacy and fewer side effects.

In this study, Hu et al. [1] examined the binding of two clinically relevant drugs, bupropion and ziprasidone, which have mechanisms of action different from those of SSRIs and SNRIs. Bupropion, an atypical antidepressant, selectively inhibits NET and DAT without affecting SERT, thus circumventing serotonergic side effects, such as sexual dysfunction and weight gain. Structural analysis revealed that bupropion stabilized NET in the inward‐facing conformation, in contrast to the effect of the previously studied DAT and SERT inhibitors. Ziprasidone, an antipsychotic with moderate affinity for NET, DAT, and SERT, features a unique chemical framework compared with that of SSRIs and SNRIs. Structural results showed that ziprasidone is also bound to NET in the inward‐facing conformation (Figure 1B). A comparative analysis between the bupropion and ziprasidone‐bound NET structures suggests that the bulky *tert*‐butylamine group of bupropion is accommodated by a distinct structural arrangement in NET, which is not feasible in SERT because of steric clashes. In contrast, the smaller ethyl group of ziprasidone allows binding across all three transporters. These findings provide a foundation for the design of new inhibitors with tailored selectivity profiles.

Recently, several studies have also reported NET structures in complexes with various inhibitors and substrates [3, 4, 5], each offering unique insights into transporter function and inhibition mechanisms. For example, Zhang et al. resolved the cryo‐EM structure of NET in a homodimer state [5], whereas other studies revealed NET as a monomer. This discrepancy may be attributed to differences in the NET constructs used or variations in the lipid composition employed for nanodisc reconstitution. Ji et al. uncovered the binding pose of radafaxine [4], a potent metabolite of bupropion, while studies by Hu et al. [1] and Tan et al. [3] elucidated the binding pose of bupropion itself. Together, these studies systematically illustrate the structural basis of bupropion and its metabolite interactions with NET, offering a comprehensive understanding of their inhibitory mechanisms. In addition, the study by Tan et al. not only identified the secondary binding site for noradrenaline but also revealed that dopamine can occupy this site. This finding, along with the insights from Hu et al., collectively underscores the functional role of the secondary substrate binding pocket in NET.

In conclusion, this study provides a detailed structural framework for NET in both the transport and inhibitory states. By elucidating the binding modes of various ligands and the conformational dynamics during the transport cycle, Hu et al. significantly advanced our understanding of the molecular mechanisms of NET. This knowledge would facilitate the development of novel therapeutics with improved efficacy and reduced side effects for the treatment of mental health disorders.

## Author Contributions

M.L. and P.S. wrote the manuscript and prepared the figure. F.Z. provided valuable discussion. All authors have read and approved the article.

## Ethics Statement

The authors have nothing to report.

## Conflicts of Interest

The authors declare no conflicts of interest.

## Data Availability

The authors have nothing to report.
